# Efficacy of botulinum toxin in the treatment of bruxism: Systematic review

**DOI:** 10.4317/medoral.22923

**Published:** 2019-06-28

**Authors:** Tania Fernández-Núñez, Sara Amghar-Maach, Cosme Gay-Escoda

**Affiliations:** 1 DDS. Student of Master’s Degree Program in Oral Surgery and Implantology (EHFRE International University/FUCSO, Belize City, Belize); 2 DDS, Resident of Master’s Degree Program in Oral Surgery and Implantology, Faculty of Medicine and Health Sciences, University of Barcelona, Barcelona, Spain; 3 MD, DDS, MS, PhD, EBOS, OMFS, Chairman and Professor of Oral and Maxillofacial Surgery, Faculty of Dentistry, University of Barcelona. Director of Master’s Degree Program in Oral Surgery and Implantology (EHFRE International University/FUCSO, Belize City, Belize). Coordinator/Researcher of the IDIBELL Institute. Head of Oral and Maxillofacial Surgery Department of the Teknon Medical Center, Barcelona, Spain

## Abstract

**Background:**

Currently it has been shown that botulinum toxin is effective for a wide variety of medical conditions, and can be applied for therapeutic purposes as cosmetic. In recent years, there has been a growing trend in the use of this drug substance to control the muscular overactivity of bruxism. The objective of this study was the use of botulinum toxin type A (BTX-A) than traditional methods, by conducting a systematic review of randomized clinical trials (RCTs) published in the health sciences literature.

**Material and Methods:**

An electronic search was made in the databases of the PubMed, Cochrane Library and Scopus data between March and October 2017, ECA, which will analyze the effect of botulinum toxin in the treatment of bruxism. We included studies of bruxist patients older than 18 years where BTX-A tests were performed on the masseter and / or temporal muscles and the control systems were injections of placebo (saline) or the use of traditional methods for the treatment of bruxism. such as occlusal splints, other medications or cognitive-behavioral therapy.

**Results:**

Of the 68 studies identified, 4 RCTs that fit our inclusion criteria were selected. These studies show that BTX-A injections can reduce the frequency of bruxism episodes, decrease pain levels and maximum occlusal force generated by this pathology, offer superior efficacy in the treatment of bruxism compared to control groups who were treated with placebo or with traditional methods for the treatment of bruxism.

**Conclusions:**

Infiltrations with BTX-A are a safe and effective treatment for patients with bruxism, so its use is justified in daily clinical practice, especially in patients diagnosed with severe bruxism.

** Key words:**Bruxism, botulinum toxins.

## Introduction

Bruxism is a repetitive activity of the masticatory muscles characterized by tightening or grinding of the teeth and which may have two distinct manifestations: sleep bruxism (SB) or awake bruxism (AB) (1). It is a common condition with an adult prevalence ranging between 8 and 31% (2) and which has acquired considerable clinical relevance due to its association with tooth abrasions and mobility, fracture of dental restorations, hypertrophy of the masseter muscle and myalgia or arthralgia characteristic of temporomandibular disorders (TMD), among other signs and symptoms (3-5). Although etiological factors have been proposed, such as emotional stress, neurological disorders, certain drugs and occlusal interferences (6,7), the etiology and pathophysiology of bruxism are still unclear, although it seems to have a multifactorial origin mediated by nervous systems: central and autonomous (8,9).

Various modalities of treatment for the management of bruxism have been investigated, such as: occlusal splints, drugs such as benzodiazepine or L-dopa and cognitive-behavioral therapy, but they have not been shown to be completely effective, since their effect does not seem to solve the cause of it and serves mainly for the management of the signs and symptoms of patients, helping to limit the destructive effects of bruxism on anatomical structures (10,11).

At present, it has been shown that botulinum toxin is effective for a wide variety of medical pathologies, used both for its therapeutic effect and for aesthetic medicine; It is a neurotoxin produced by a Gram-positive aerobic bacterium called Clostridium botulinum. There are seven different types of exotoxins, botulinum toxin type A (BTX-A) is a biological variant that temporarily inhibits skeletal muscle by hindering the production of acetylcholine and inactivating calcium channels in nerve endings. In recent years, there has been an increasing trend in the use of this drug to control the activity of bruxism (12).

The aim of this study is included in the following PICO question: in bruxist patients, is the use of BTX-A more effective than the traditional methods used up to now on the signs and symptoms of bruxism? To answer this question, a systematic review of randomized clinical trials (RCTs) found in the health sciences literature was conducted.

## Material and Methods

The planning and preparation of this study has followed the guidelines established by the PRISMA declaration (13) for the preparation of systematic reviews and meta-analysis.

-Eligibility criteria

We included randomized controlled clinical trials (RCTs) involving bruxist patients older than 18 years in which the effect of botulinum toxin in the treatment of bruxism compared with traditional therapy is analyzed. Studies in which bruxism was caused by psychological or neurological disorders and those who used this therapy aimed at the treatment of other diseases were excluded.

The test interventions were injections of botulinum toxin type A (BTX-A) in the masseter and/or temporal muscles and the control interventions were injections of placebo (saline solution) or the use of traditional methods for the treatment of bruxism such as occlusal splints, medications or cognitive-behavioral therapy.

-Search strategy

A search of articles was carried out in the PubMed, Cochrane Library and Scopus databases between March and October 2017, using the keywords “bruxism” and “botulinum toxins” combined with the Boolean operator “AND” to obtain the articles that included the search terms used (“bruxism” [MeSH Terms] AND “botulinum toxins” [MeSH Terms]) in all databases.

-Study selection

Studies that were published in English from 2007 to 2017 and that included at least 10 patients were selected. Case series and animal studies were excluded. The selection of the articles was carried out by consensus between two of the authors (TFN and SAM) based on the inclusion and exclusion criteria established. Their level of scientific evidence was also taken into account according to the principles of the SIGN (Scottish Intercollegiate Guidelines Network) (14) and only articles classified in the first two levels were selected (meta-analysis, systematic reviews of clinical trials and high-quality clinical trials with very low or low risk of bias) and studies of low evidence were discarded.

-Risk of bias in individual studies 

To evaluate the reliability of the results of the selected studies, the Cochrane criteria for the risk assessment of bias (version 5.1.0) (15) were used, through which the following was analyzed: sequence generation, allocation concealment, blinding of participants, incomplete outcome data, selective outcome reporting, as well as other possible sources of bias such as conflict of interests, in each of the included studies.

-Statistical analysis

A meta-analysis will be implemented if the results of the studies are comparable, that is, when the same outcome variables were measured with a similar technique.

## Results

-Study selection and description 

**Figure 1:**
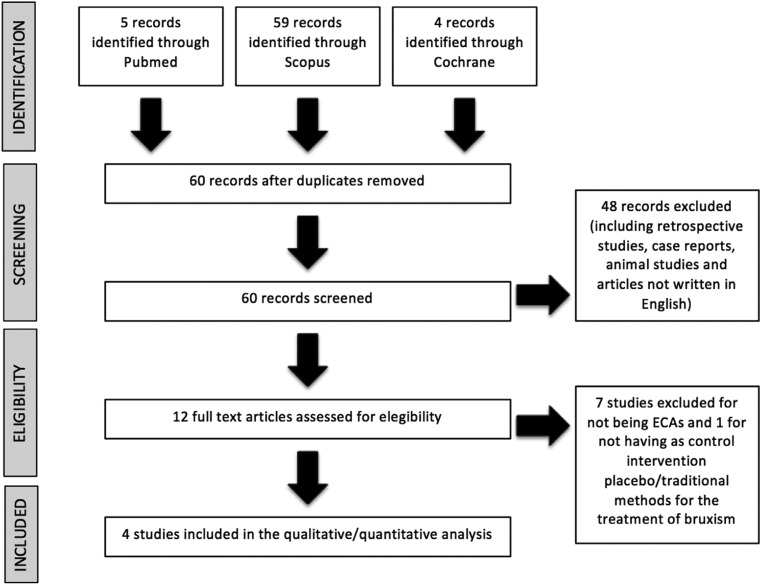
Flow chart of the article. Selection process for the systematic review, according to PRISMA guidelines (13).

In the initial search, a total of 68 articles were obtained; 5 in PubMed, 4 in Cochrane Library and 59 in Scopus, of which 4 studies met our inclusion criteria as reflected in the PRISMA flow chart (Fig. 1). These studies were classified according to their level of scientific evidence and grade of recommendation using the SIGN (Scottish Intercollegiate Guidelines Network) guidelines. Two of the studies (16,17) showed a level of evidence 1- because it has a high risk of bias; On the other hand, the other two (18,19) showed a level of scientific evidence 1+ and a grade of recommendation B.

The main characteristics of the studies included in the present systematic review are shown in  Table 1.

**Table 1:**
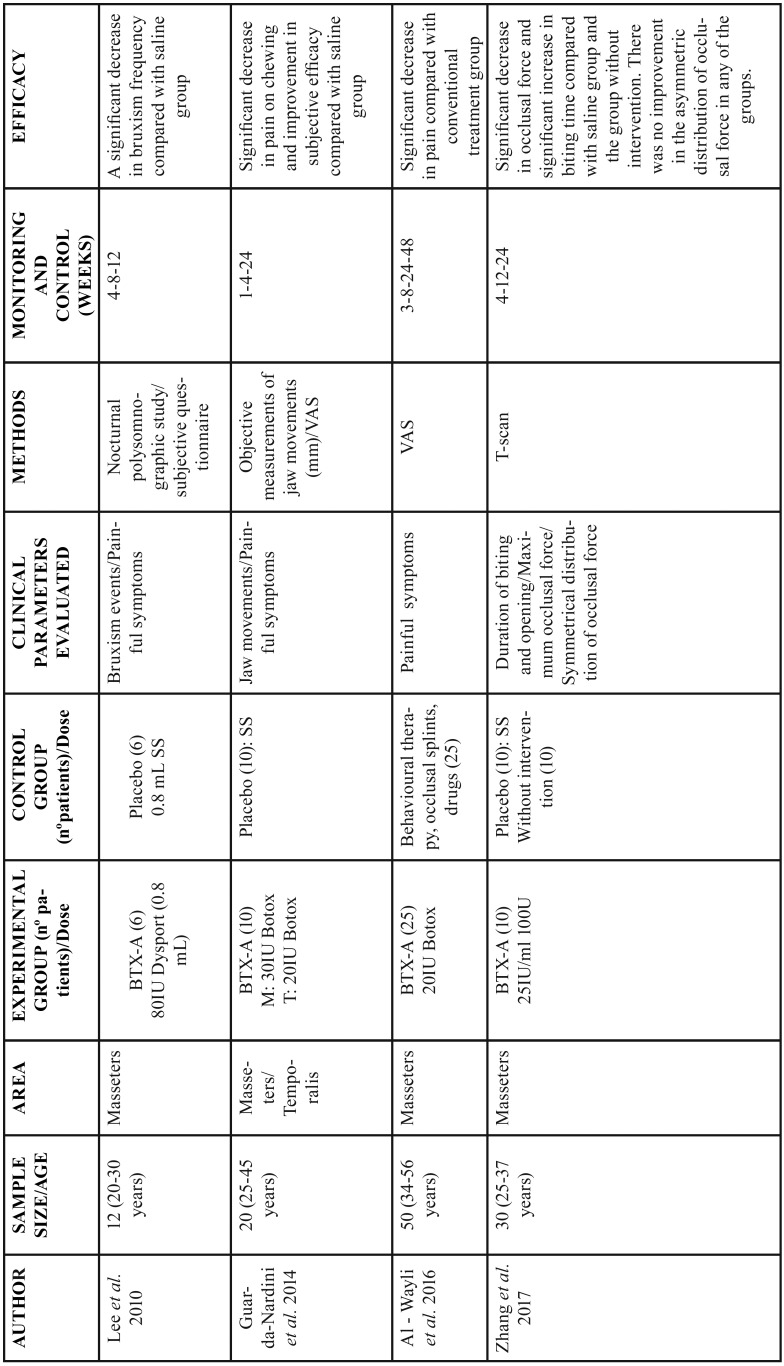
General information of the included studies. BTX-A: botulinum toxin A; IU: international units; M: masseter; T: temporalis; SS: saline solution; VAS: visual analog scale.

Lee *et al.* (18) performed a nocturnal electromyographic recording of the masseter and temporal muscles in 12 patients with bruxism, six of them received infiltrations of 80 IU of BTX-A (Dysport®, Ipsen Pharma, Wrexham, United Kingdom) distributed in three points of both masseter muscles, and the other six an equivalent amount of saline. The patients who received BTX-A reported a clinical improvement of the pathology; it was observed that the number of bruxism events decreased significantly after the injection of botulinum toxin in the masseter muscle but not in the temporalis muscle, without differing in the three post-injection times. Regarding the bruxism symptoms questionnaire data, the score decreased after the intervention in both the BTX-A and in the saline injection groups ( Table 2).

**Table 2:**
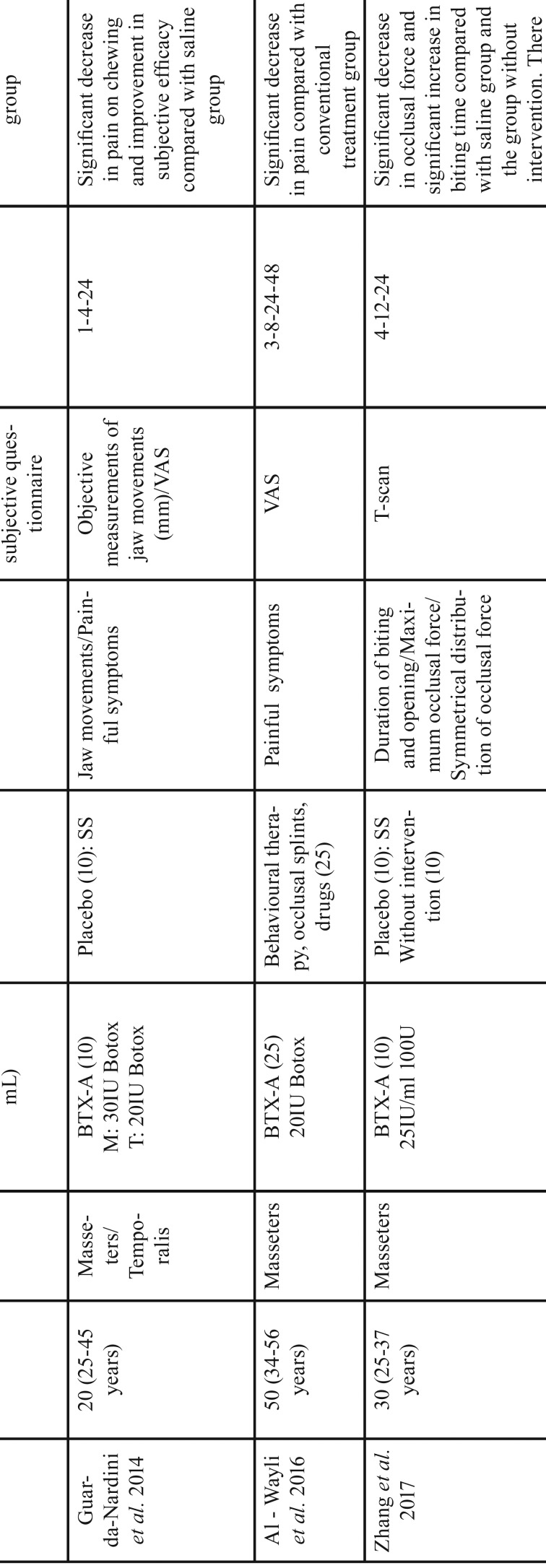
Results of the clinical parameters evaluated by each study. BTX-A: botulinum toxin A; SS: saline solution; B: baseline; w: weeks; mm: millimeters; VAS: visual analog scale; TMTB: traditional methods of treating bruxism; sec: seconds; C: control group without intervention.

In the study by Guarda-Nardini *et al.* (19) injected 30 IU of BTX-A (Botox®, Allergan Inc., Irvine, E.E.U.U) in masseter muscles and 20 IU in temporal muscles to 10 patients with myofascial pain associated with bruxism. The descriptive analysis of the study showed a slight increase in the values of the maximum unassisted and assisted movements of buccal, protrusive and laterotrusive opening (mm) in the BTX-A group (the differences between the baseline and follow-up values had a tendency to increase) and remained unchanged in the placebo group. Regarding the symptoms (pain at rest and in mastication), evaluated by means of a visual analogue scale (VAS), they decreased in the BTX-A group while in the placebo group it remained constant, although the effectiveness of the mastication did not improve in neither of the two groups ( Table 2).

Al-Wayli *et al.* (16) compared the efficacy of treatment with BTX-A versus conventional treatments for bruxism. For this, they recruited 50 subjects diagnosed with nocturnal bruxism, 25 were infiltrated with 20 IU of BTX-A (Botox®, Allergan Inc., Irvine, USA) in three points of both masseters, and the other 25 were treated with behavioral therapy, occlusal splint and pharmacological measures (diclofenac 50 mg). In the assessment of pain, there were no significant differences in the mean preoperative pain score between the two groups, however, there was a relevant difference in the average value of postoperative pain at 3 weeks in both the BTX-A group and in the control group that used traditional methods for the treatment of bruxism. From the second postoperative month, the mean pain score decreased significantly in both groups, reaching its lowest value at the sixth month and being much lower in the BTX-A group than in the control group ( Table 2).

Zhang *et al.* (17) recruited 30 patients with bruxism to assess the therapeutic efficacy of BTX-A injection in terms of occlusal force, biting time and symmetric distribution of occlusal force using a T-Scan. 10 patients were infiltrated with 25 IU/ml of BTX-A in three points of both masseter muscles, another 10 were infiltrated with saline in the same way and the remaining 10 were a control group without any intervention. The bite time in the BTX-A group was significantly longer after three months, and decreased after six months, but there were no significant changes in the placebo and control groups. With respect to the maximum occlusal force, significantly lower values were achieved in the BTX-A group compared to the other two groups where there were no relevant differences, reaching its lowest value after three months of treatment. There were no significant differences between the groups in the symmetric distribution of the occlusal force, however, there was a tendency towards its improvement during the treatment ( Table 2).

-Risk of bias assessment 

**Figure 2:**
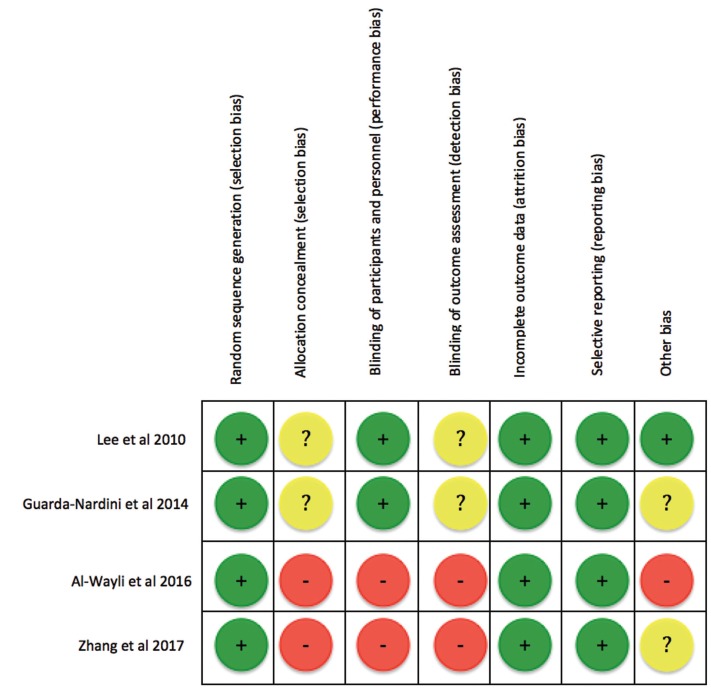
Risk of bias assessment. According to the Cochrane Hand book for Systematic Reviews of Interventions, version 5.1.0 (15). +: low risk of bias; - : high risk of bias; ?: unclear risk of bias.

The evaluation of the risk of bias through the Cochrane criteria showed positive results in the generation of random sequence, and in the absence of incomplete data and selective results for the four included studies, but in none of them, despite being RCTs , the method used to generate the allocation sequence and the measures to blind the participants and the study staff, in particular the studies of Zhang *et al.* (17) and Al-Wayli *et al.* (16) they do not specify that they be blinded, and in this last one the sample was formed entirely by women, this makes that these last two studies have a high risk of bias, and those of Lee *et al.* (18) and Guarda-Nardini *et al.* (19) a low risk of bias (Fig. 2).

-Data extraction: qualitative synthesis 

The results of these studies showed that BTX-A injections can reduce the frequency of bruxism episodes, decrease pain levels and the maximum occlusal force generated by this pathology, offering superior effectiveness in the treatment of bruxism compared to the control groups that were treated with placebo or with traditional methods for the treatment of bruxism. Of the four included studies, Guarda-Nardini *et al.* (19) and Al-Wayli *et al.* (16), did not specify if the botulinum toxin injections had adverse effects, however Lee *et al.* (18) and Zhang *et al.* (17) reported the absence of such adverse effects, both locally and systemically.

-Data extraction: quantitative synthesis 

Due to the heterogeneity presented to the results of the different studies, since each one measures the variables in different ways, it is not possible to do a meta-analysis with the available data.

## Discussion

Botulinum toxins, purified exotoxins of Clostridium botulinum, have been used for a long period of time for numerous neuromuscular disorders (20,21). These toxins can inhibit neuromuscular transmission, which justifies its clinical application in the treatment of bruxism, since recent scientific evidence has indicated that bruxism has a multifactorial etiology mediated by the central nervous and autonomic systems, which regulate the motor activity of the chewing muscles (22,23).

Currently, many authors (24-26) support the use of BTX-A for the treatment of various conditions of the oral-maxillofacial region based on the positive results obtained in various clinical trials collected in the literature. Rao *et al.* (24) made a review showing the results of several clinical trials and case reports that supported the use of BTX-A for the treatment of TMD, gingival smile correction, muscle hypertrophy and spasms, headache (migraine), trigeminal neuralgia and even after the placement of dental implants. They noted that although BTX-A could decrease muscle strength and mastication, it is temporary and normal function would return when the effect of the toxin disappeared. Gay-Escoda *et al.* (25) conducted a review of the literature that included clinical trials in which BTX-A was used in the salivary glands for the treatment of sialorrhea derived from different neurological disorders such as infantile cerebral palsy, the disease of Parkinson’s and amyotrophic lateral sclerosis. More than half of the authors injected the product into the parotid glands, 9.5% in the submaxillary glands and 38% in both. The total doses of toxin injected varied from 10 to 100 IU of Botox® or 30 to 450 IU of Dysport® according to the different authors. A reduction in saliva production was observed after these injections, and the duration of the therapeutic effect was 1.5-6 months. Six articles (30%) described the presence of adverse effects such as dysphagia, xerostomia and difficulties to chew. The authors concluded that the injection of BTX-A in the salivary glands may be a valid treatment option in patients with sialorrhea, since it is able to improve the quality of life, however, it is important to be aware that the duration of the therapeutic effect is limited in time and usually lasts a few months. In a retrospective study carried out by Alonso-Navarro *et al.* (26), the evolution of 19 patients with severe bruxism who were treated periodically with infiltrations of BTX-A in both temporal and masseter muscles, using initial doses of 25 IU per muscle, during follow-up periods of 0.5 to 11 was described; the doses were adjusted throughout the follow-up according to the degree of response observed. None of the patients presented side effects. The final dose ranges reached ranged from 25 to 40 IU per muscle and the duration of the effects ranged from 13 to 26 weeks. Based on these results, they concluded that infiltrations with BTX-A are a safe and effective treatment for patients with severe bruxism. The four studies that we have selected for the review showed that BTX-A injections can reduce the frequency of bruxism episodes, decrease pain levels and the maximum occlusal force generated by this pathology, offering more satisfactory clinical results in the treatment of bruxism compared to control groups that were treated with placebo or traditional methods, thus reiterating that the use of BTX-A is an effective alternative in the treatment of this pathology.

Psychological factors play a very important role both in the etiology and in the treatment of bruxism (27). This aspect is reflected in the results of the study by Zhang *et al.* (17), since the values of maximum masticatory force had been reduced, although to different degrees, both in the BTX-A experimental group, and in the placebo and control groups without intervention. This change in biting force in the placebo and control groups indicates that psychological intervention can play an important role in the treatment of bruxism.

The effect of the BTX-A is transient and is largely limited to the injection area. A review by Ihde and Konstantinovic (28) has indicated that the most common adverse effects of BTX-A are local, such as sensitivity and mild cutaneous reaction at the site of injection, systemic effects, such as headache and nervous atrophy. reversible, and specific effects, including dysphonia, dysphagia and dry mouth. However, of the four studies included in the review, only two (Lee *et al.* (18) and Zhang *et al.* (17)) reported the absence of adverse effects at the time of treatment, but none of them reported the possible adverse effects after the injection. Tan and cols. (29) and Monroy *et al.* (30) have explained in their studies that the use of BTX-A in the treatment of bruxism can cause dysphagia or mild pain at the site of injection and temporary drooling, however, patients who suffered these adverse effects received a large dosage (> 100 IU) or had a condition complicated by other medical conditions. In none of the four studies we have selected exceeds the total dose of 100 IU, therefore, injections of BTX-A at a dose lower than 100 IU in the masseter or temporal muscles in patients who are otherwise healthy are safe, its use being feasible in the usual clinical practice.

The results of the present review showed that injections of BTX-A in the masseter and/or temporal muscles can be a valid treatment option in patients with bruxism, since they can improve the quality of life. However, it is not exempt from limitations, since most of the samples are too small to allow obtaining statistically significant results. Furthermore, in none of the included studies, despite being randomized clinical trials, the method used to generate the allocation sequence is described, nor are the measures to blind the participants and the study staff, in fact, in two of the studies do not specify that they be blinded and in one of them the sample completely lacked homogeneity since it was completely made up of women. To all this, it must be added that in all the studies except one, the follow-up period for the patients was less than one year, which makes the need for long-term controlled studies that allow us to assess both the therapeutic effect indispensable. as the adverse effects of this treatment over the years.

## Conclusions

In conclusion, the infiltrations of BTX-A can reduce the frequency of bruxism episodes, as well as the masticatory force, and decrease the levels of pain derived from it, which translates into an improvement in the quality of life of patients. In addition, in doses <100UI it is a safe treatment with a low probability of adverse effects occurring in healthy patients. Therefore, the use of BTX-A is a safe and effective treatment for patients with bruxism that shows better clinical results than traditional methods such as occlusal splints, drugs or cognitive-behavioral therapy, so its use would be justified in daily clinical practice, especially in patients diagnosed with severe bruxism.
